# Trends in sepsis-associated cardiovascular disease mortality in the United States, 1999 to 2022

**DOI:** 10.3389/fcvm.2024.1505905

**Published:** 2024-12-09

**Authors:** Malik Salman, Jack Cicin, Ali Bin Abdul Jabbar, Ahmed El-shaer, Abubakar Tauseef, Noureen Asghar, Mohsin Mirza, Ahmed Aboeata

**Affiliations:** ^1^ School of Medicine, Creighton University, Omaha, NE, United States; ^2^ Department of Cardiology, Creighton University Medical Center, Omaha, NE, United States

**Keywords:** sepsis, cardiovascualr disease, COVID-19, CDC WONDER database, mortality trends

## Abstract

**Purpose:**

Cardiovascular disease (CVD) is the leading cause of death in the United States, and sepsis significantly contributes to hospitalization and mortality. This study aims to assess the trends of sepsis-associated CVD mortality rates and variations in mortality based on demographics and regions in the US.

**Methods:**

The Centers for Disease Control and Prevention Wide-ranging Online Data for Epidemiologic Research (CDC WONDER) database was used to identify CVD and sepsis-related deaths from 1999 to 2022. Data on gender, race and ethnicity, age groups, region, and state classification were statistically analyzed to obtain crude and age-adjusted mortality rates (AAMR). The Joinpoint Regression Program was used to determine trends in mortality within the study period.

**Results:**

During the study period, there were a total of 1,842,641 deaths with both CVD and sepsis listed as a cause of death. Sepsis-associated CVD mortality decreased between 1999 and 2013, from AAMR of 65.7 in 1999 to 58.8 in 2013 (APC −1.06*%, 95% CI: −2.12% to −0.26%), then rose to 74.3 in 2022 (APC 3.23*%, 95% CI: 2.18%–5.40%). Throughout the study period, mortality rates were highest in men, NH Black adults, and elderly adults (65+ years old). The Northeast region, which had the highest mortality rate in the initial part of the study period, was the only region to see a decline in mortality, while the Northwest, Midwest, and Southern regions experienced significant increases in mortality rates.

**Conclusion:**

Sepsis-associated CVD mortality has increased in the US over the past decade, and both this general trend and the demographic disparities have worsened since the onset of the COVID-19 pandemic.

## Introduction

Cardiovascular disease (CVD) is the leading cause of death in the United States, comprising 26.9% of annual mortality across all age groups (1). Sepsis contributes to at least 1.7 million hospitalizations of US adults each year, and conservative estimates place the cost of sepsis to the US healthcare system at $62 billion annually (2, 3). Concerningly, despite heightened attention to the mortality burden of sepsis, mortality rates in people aged 65 and over crept upwards from 277 per 100,000 in 2019 to 331 per 100,000 in 2021 (4, 5). Septic patients with cardiovascular dysfunction have been shown to have an increased mortality rate of 70%–90%, as opposed to 20% in those without cardiovascular impairment (6). Sepsis has also been shown to increase the likelihood of atherosclerotic and nonatherosclerotic cardiovascular events—both in-hospital and after hospital discharge—increasing the risk of mortality of sepsis patients (7–9). Though the precise pathophysiology of myocardial dysfunction in sepsis remains unclear, hemodynamic compromise and cytokine release are known to play a role in myocardial depression and ischemia (10–12).

With the COVID-19 pandemic came an acute rise in cardiovascular disease-related deaths, from 874,613 in 2019 to 928,741 in 2020 (13). The pandemic is thought to have increased CVD mortality due to the implications of viral infection and its effect on healthcare delivery as a whole. At the same time, sepsis-related deaths increased by nearly 36,000 beyond the expected rise in 2020 (14). Given the pathophysiological interplay between CVD and sepsis, it is crucial to determine the difference in the mortality rates for various demographic (i.e., racial and ethnic groups, gender, and age groups) and regional groups, long-term mortality trends, and how the recent COVID-19 pandemic has impacted these trends. Hence, to further understand these differences, we utilized the Centers for Disease Control and Prevention Wide-Ranging Online Data for Epidemiologic Research (CDC WONDER) national database to analyze the death records of patients with both CVD and sepsis-related mortality in the United States from 1999 to 2022.

## Methods

### Study design and database

Centers for Disease Control and Prevention Wide-ranging Online Data for Epidemiologic Research (CDC WONDER) was used to identify CVD and septicemia-related deaths in the United States. The Multiple Cause of Death public use record and the CDC WONDER database death certificate records were analyzed to determine the CVD and septicemia-related cause of death as an underlying or contributing cause on nationwide death certificate records. The study was exempt from institutional review board approval because the CDC WONDER database contains anonymized, publicly available data. We extracted data regarding CVD and septicemia-related deaths and population sizes from 1999 to 2022. The following International Classification of Diseases (co), 10th Revision, Clinical Modification codes were used to identify deaths from CVD and Septicemia- I00-I78 for Major cardiovascular diseases; A40-A41 for Septicemia.

### Demographic and geographical study groups

Specifically, data extracted for analysis included gender, race and ethnicity, age groups, region, and state. Genders included males and females. Race and ethnicity groups were divided into non-Hispanic (NH) white, NH Black persons or African American, NH American Indian or Alaska Native, and Hispanic or Latino. Age groups included adults aged 45–64 (middle-aged group) and those aged over 65 (elderly group). We only included middle-aged and elderly populations in our analysis because the majority of deaths due to CVD and sepsis happen in these age groups. We extracted data for four Census Regions (Northeast, Midwest, South, and West) as classified by the Census Bureau definitions. Statistical analysis of CVD and septicemia-related crude mortality rate (CMR) and age-adjusted mortality rates (AAMR) was performed. Crude mortality rates were calculated by dividing the number of CVD and sepsis-related deaths by the corresponding United States population. The age-adjusted mortality rate (AAMR) accounts for variations in age distribution, enabling comparison of data. The United States population as of the year 2000 was used as the standard population for determining AAMR. The Joinpoint Regression Program (Joinpoint version 4.9.0.0, available from the National Cancer Institute in Bethesda, Maryland) was used to analyze trends in mortality over the study period. This program identifies significant changes in annual mortality trends over time through Joinpoint regression, fitting models of linear segments where significant temporal variation occurred. Annual percentage change (APC) and 95% confidence intervals (CIs) for the AAMRs were calculated for the line segments linking Joinpoints using the Monte Carlo permutation test. The weighted average of the APCs was calculated and reported as average annual percent change (AAPC), along with corresponding 95% CIs to summarize the reported mortality trend for the entire study period. A 2-tailed *t*-test was used to determine if the APC and AAPCs indicated an increase or decrease in mortality over the time interval. Statistical significance was evaluated at *p* ≤ 0.05 and is represented in our results, figures, and Supplementary Material.

## Results

From 1999 to 2022, there were a total of 33,836,679 deaths related to CVD, 4,019,524 deaths related to sepsis, and 1,842,641 deaths with both cardiovascular disease and sepsis listed as a cause of death. Of these, 935,073 (50.74%) were men and 907,568 (49.25%) were women. 1,328,867 deaths (72.12%) were in Non-Hispanic (NH) White adults, 289,271 (15.70%) were NH Black adults, 151,793(8.24%) were Hispanic adults, and 11,873 (0.64%) were NH American Indian or Alaskan Native adults. 346,165 (18.79%) of the deaths were in middle-aged adults (45–64 years old) and 1,496,476 (81.21%) were in elderly adults (65+ years old).

**Figure 1:**
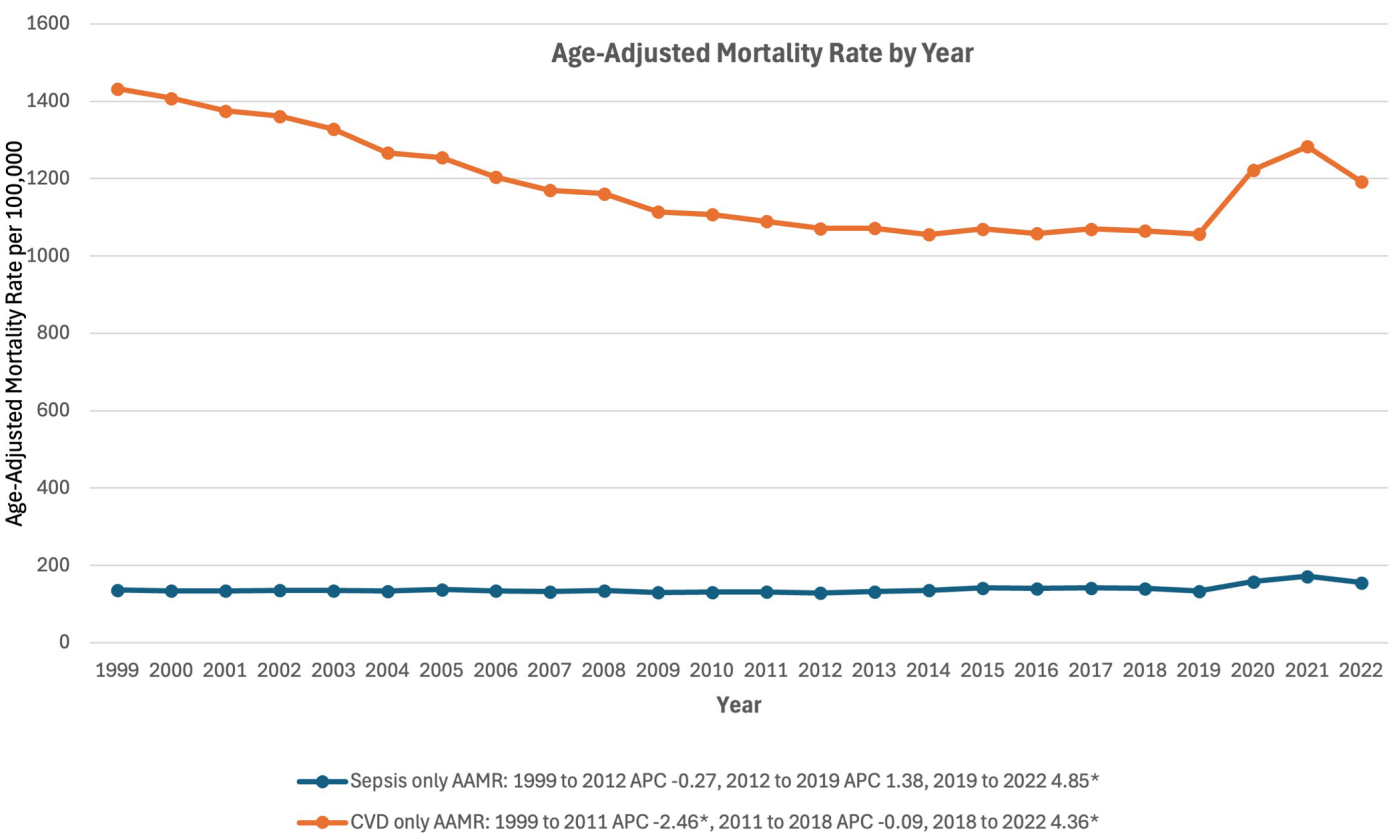
Trends in mortality rates associated with separate sepsis and cardiovascular disease related mortality in the United States between 1999 and 2020. *Indicates the APC is significantly different from 0.

Overall, there was a significant decline in mortality due to CVD from AAMR of 1,432.56 in 1999 to 1191.99 in 2022 (AAPC −0.58%, 95% CI: −0.87% to −0.34%). During this time, AAMR for CVD initially declined between 1999 and 2011 (APC −2.46*% 95% CI: −4.19% to −1.94%), then stayed stable from 2011 to 2019 (APC −0.09%, 95% CI: −1.97% to 1.57%) and finally increased from 2019 to 2022 (APC 4.36*%, 95% CI: 2.15%–8.32%) (Figure 1). Compared to that, the AAMR of Sepsis stayed stable from 136.54 in 1999 to 134.04 in 2019 and then significantly increased to 172.15 by 2021 (APC 4.85*%, 95% CI: 1.44% to 9.55%) (Figure 1).

**Figure 2:**
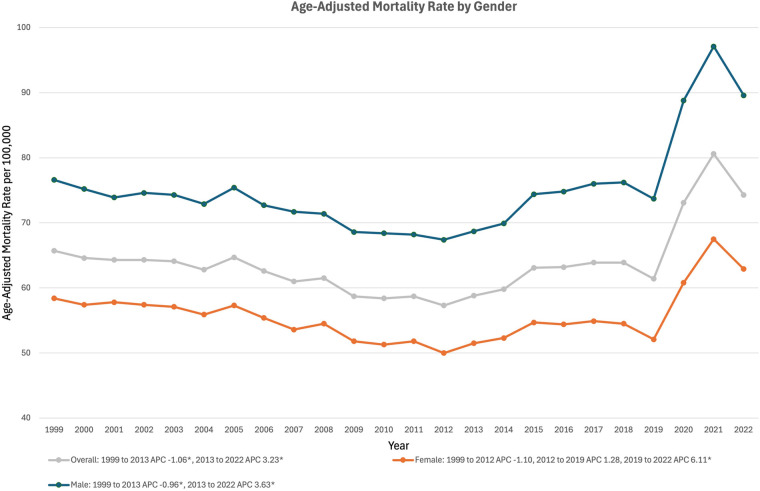
Trends in sepsis-associated cardiovascular disease mortality, overall and stratified by gender, in the United States between 1999 and 2020. *Indicates the APC is significantly different from 0.

Sepsis-related CVD mortality decreased between 1999 and 2013, from an AAMR of 65.71 in 1999 to 58.79 in 2013 (APC −1.02*%, 95% CI: −2.13% to −0.33%) (Figure 2). AAMR then rose to 74.33 in 2022 (APC 3.14*%, 95% CI: 2.01%–5.40%) (Figure 2).

### Gender stratified

In males, sepsis-associated CVD mortality dropped from an AAMR of 76.64 in 1999 to 68.72 in 2013 (APC −0.96*% 95% CI: −2.05% to −0.22%) (Figure 2). Male mortality then rose in the following years, climbing to 89.59 by 2022 (APC 3.56*%, 95% CI: 2.45% to 5.75%) (Figure 2). In females, mortality declined from an AAMR of 58.43 in 1999 to 50.00 in 2012 (APC −1.11%, 95% CI: −3.57% to 0.14%) (Figure 2). Mortality then began to rise, reaching 52.10 in 2019 (APC of 1.25%, 95% CI: −1.93% to 2.88%) and then 62.87 in 2022 (APC of 6.13*%, 95% CI: 2.59%–10.82%) (Figure 2).

### Race stratified

**Figure 3:**
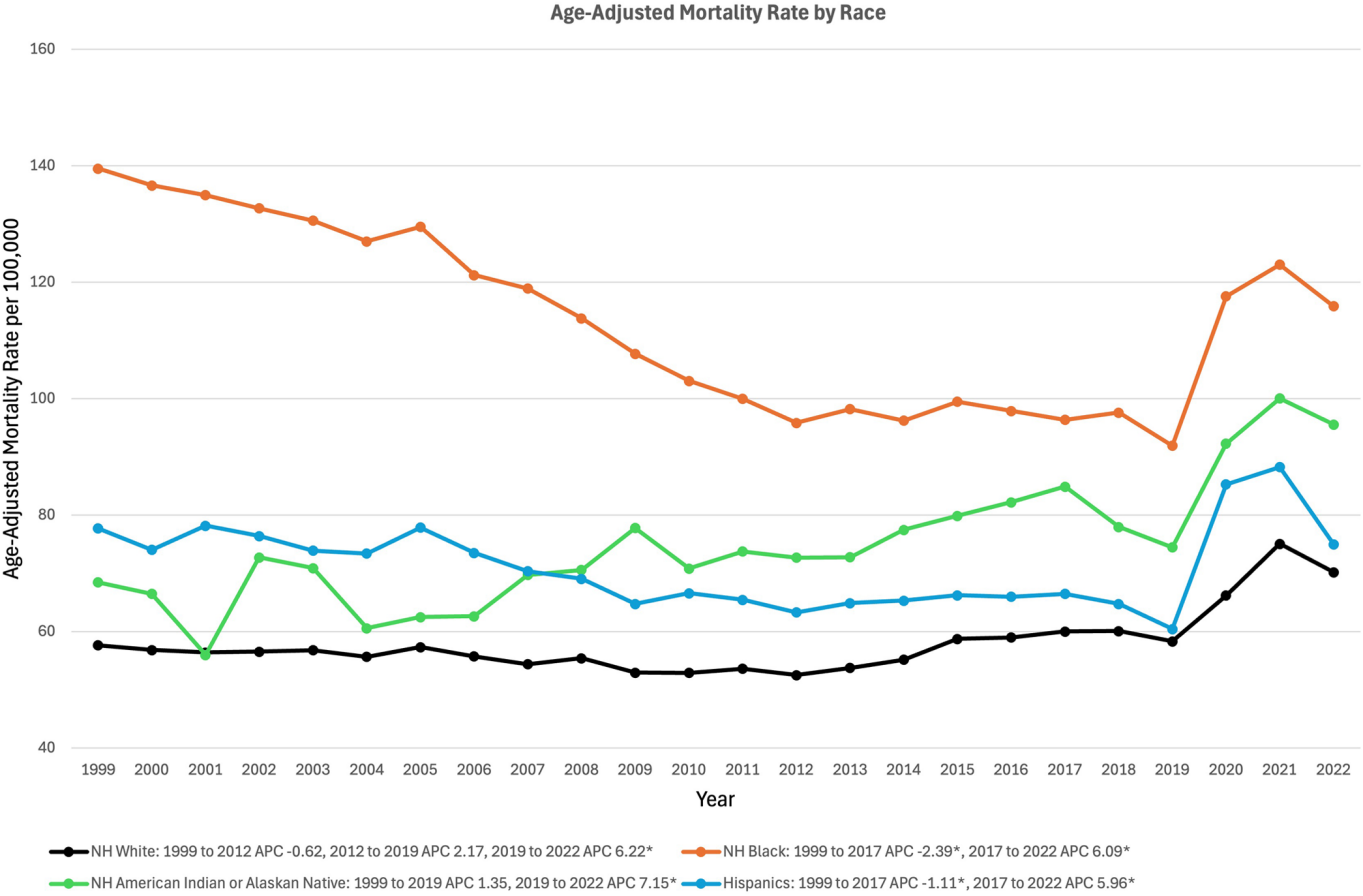
Trends in sepsis-associated cardiovascular disease mortality, stratified by race, in the United States between 1999 and 2020. *Indicates the APC is significantly different from 0.

In White adults, AAMR decreased from 57.62 in 1999 to 52.50 in 2012 (APC −0.62%, 95% CI: −2.67% to 0.79%), increased to 58.31 in 2019 (APC 2.17% 95% CI: −1.85% to 3.40%), and finally increased to 70.19 in 2022 (APC 6.22*%, 95% CI: 3.09%–10.54%) (Figure 3). In Hispanic adults, AAMR decreased from 77.72 in 1999 to 66.47 in 2017 (APC −1.11*%, 95% CI: −2.47% to −0.29%) and increased to 75.00 in 2022 (APC 5.96*%, 95% CI: 2.30% to 14.95%) (Figure 3). In American Indian or Alaskan Native adults, AAMR increased from 68.45 in 1999 to 74.45 in 2019 (APC 1.35%, 95% CI: −4.46% to 18.90%), and again increased to 95.56 in 2022 (APC 7.15*%, 95% CI: 1.18%–14.72%) (Figure 3). In Black Adults, AAMR decreased from 139.54 in 1999 to 96.38 in 2017 (APC −2.39*%, 95% CI: −3.00% to −1.88%), followed by an increase to 115.89 in 2022 (APC 6.09*%, 95% CI: 3.21% to 11.50%) (Figure 3).

### Age group stratified

**Figure 4:**
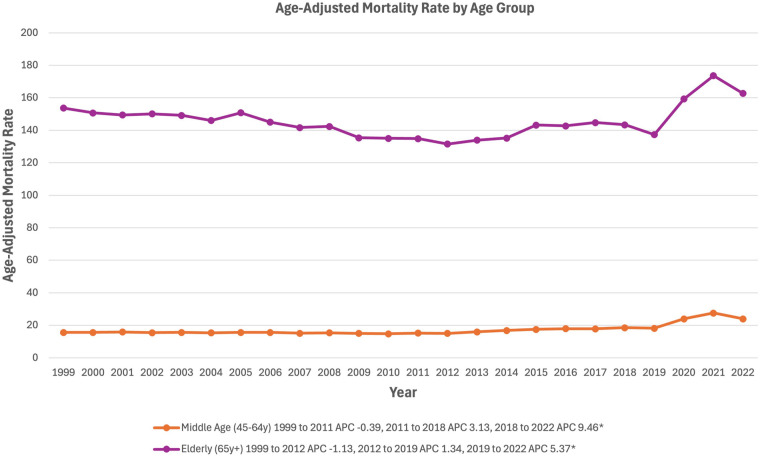
Trends in sepsis-associated cardiovascular disease mortality, stratified by age group, in the United States between 1999 and 2020. *Indicates the APC is significantly different from 0.

In middle-aged adults (45 to 64 years old) AAMR remained relatively stable, from 15.62 in 1999 to 15.27 in 2011 (APC −0.39%, 95% CI, −6.31% to 6.42%), and increased to 18.57 in 2018 (APC 3.13%, 95% CI, −3.14% to 6.69%), followed finally by a significant increase to 24.03 in 2022 (APC 9.46*%, 95% CI, 4.91% to 17.46%) (Figure 4). In elderly adults (65+ years old), AAMR decreased from 153.73 in 1999 to 131.59 in 2012 (APC −1.13%, 95% CI, −3.60% to 0.97%) (Figure 4). From 2012, elderly adult AAMR increased to 137.33 in 2019 (APC 1.34%, 95% CI, −2.52% to 2.72%), followed by a significant jump to 162.73 in 2022 (APC 5.37*%, 95% CI, 2.21%–9.56%) (Figure 4).

### Region stratified

**Figure 5:**
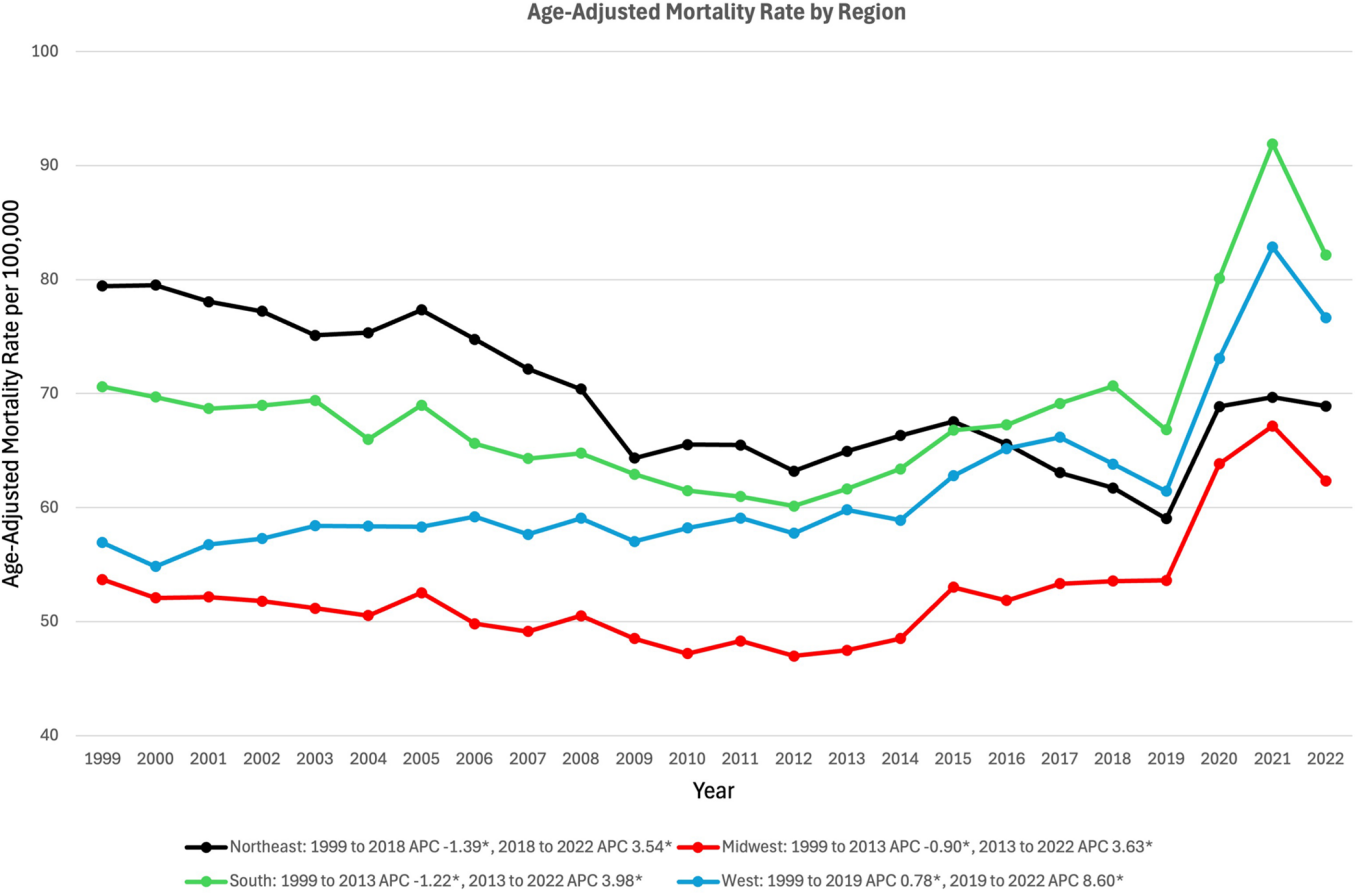
Trends in sepsis-associated cardiovascular disease mortality, stratified by geographic region, in the United States between 1999 and 2020. *Indicates the APC is significantly different from 0.

In the Northeast, AAMR decreased from 79.44 in 1999 to 61.72 in 2018 (APC −1.39*%, 95% CI: −1.81% to −1.09%), and increased to 68.91 in 2022 (APC 3.54*%, 95% CI: 0.81% to 9.13%) (Figure 5). In the Midwest, AAMR decreased from 53.70 in 1999 to 47.48 in 2013 (APC −0.90*%, 95% CI: −1.56% to −0.37%), and increased to 62.34 in 2022 (APC 3.63*%, 95% CI: 2.75%–4.93%) (Figure 5). In the South, AAMR decreased from 70.61 in 1999 to 61.65 in 2013 (APC −1.22*%, 95% CI: −2.37% to −0.43%), and increased to 82.18 in 2022 (APC 3.98*%, 95% CI: 2.77%–6.15%) (Figure 5). In the West, AAMR rose from 56.94 in 1999 to 61.44 in 2019 (APC 0.78*%, 95% CI: 0.29%–1.16%) and again increased to 76.67 in 2022 (APC 8.60*%, 95% CI: 4.06%–15.20%) (Figure 5).

### State-level stratified

**Figure 6:**
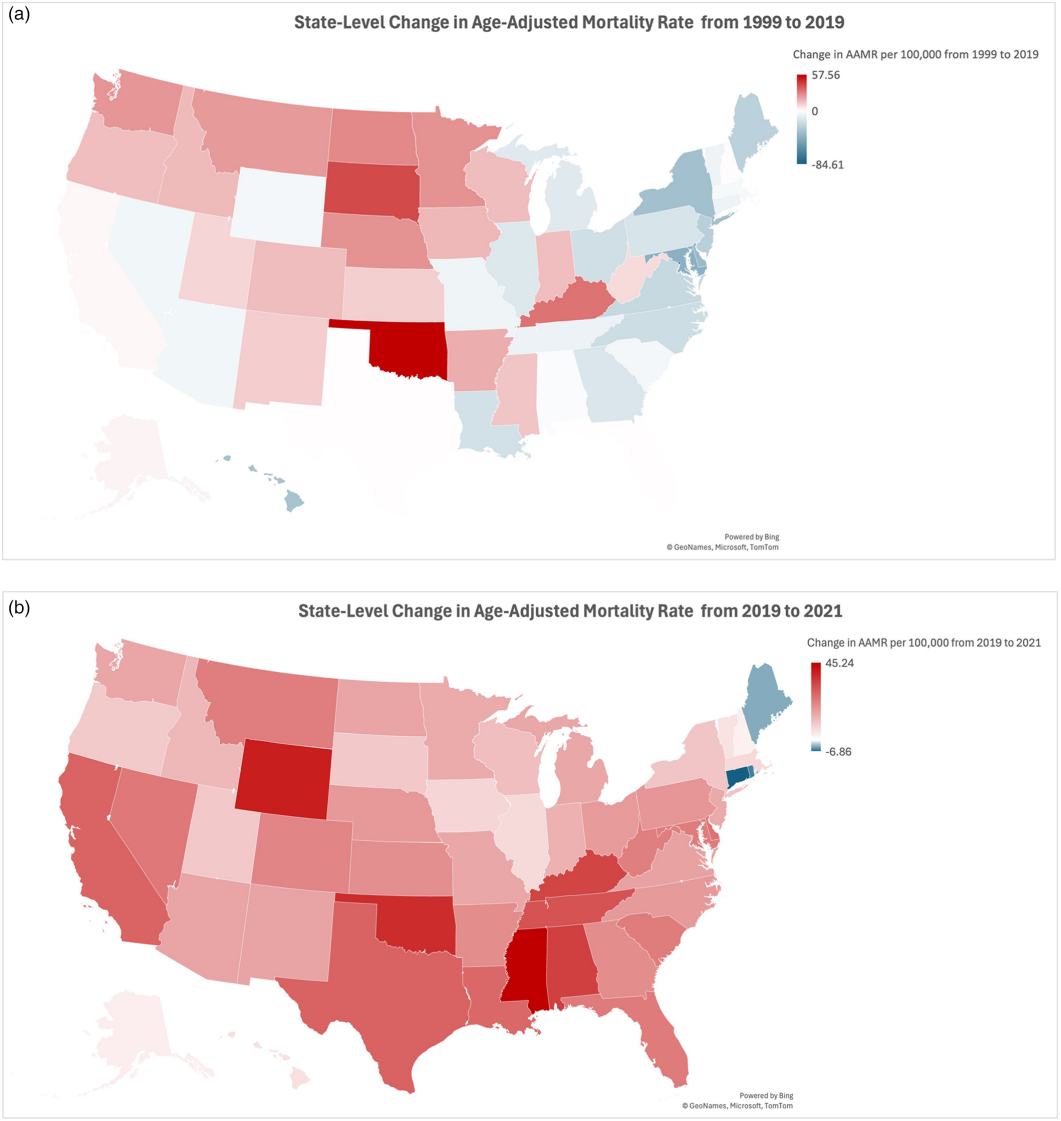
**(a)** Map showing trends in sepsis-associated cardiovascular disease mortality, stratified by state, in the United States between 1999 and 2019. **(b)** Map showing trends in sepsis-associated cardiovascular disease mortality, stratified by state, in the United States between 2019 and 2021.

An inspection of state-level change in sepsis-associated CVD age-adjusted mortality reveals the drastic AAMR increases that impacted most US states between 2019 and 2021. Between 1999 and 2019, AAMR declined in twenty-five states and the District of Columbia, while AAMR increased in the other twenty-five states. The greatest AAMR decreases during this period occurred in the District of Columbia (AAMR change of −84.61), Maryland (−41.37), Delaware (−35.96), New York (−33.73), and Hawaii (−31.22) (Figure 6a). Conversely, the greatest increases occurred in Oklahoma (AAMR change of 57.56), South Dakota (40.85), Kentucky (31.41), North Dakota (26.66), and Nebraska (24.71) (Figure 6a). Between 2019 and 2021, sepsis-associated CVD mortality rose in all but three states: Connecticut (−6.86), Rhode Island (−5.48), and Maine (−3.58) (Figure 6b). Of the remaining forty-seven states and the District of Columbia where AAMR increased, the greatest increases occurred in Mississippi (45.24), Wyoming (39.99), Oklahoma (37.24), Alabama (33.87), and Kentucky (32.92) (Figure 6b).

## Discussion

Our analysis of 23 years of mortality data collected from the CDC demonstrates the increased AAMR from sepsis-associated cardiovascular disease following the COVID-19 pandemic. The study of mortality data concluded that males had a higher sepsis-associated CVD mortality than females. NH Black adults had the highest mortality rates throughout the study period as compared to all other races, and elderly adults over the age of 65 years had the highest mortality amongst all age groups. Out of the geographical regions, the Northeast was the only region to see a decline in mortality over the study period. Oklahoma was the state with the greatest increase in AAMR from 1999 to 2019, while Mississippi had the greatest increase in AAMR from 2019 to 2021.

From 1999 to 2013, AAMR declined at a relatively stable rate for both men and women, after which it began to rise slowly before drastically increasing in 2019 with the onset of the pandemic. The massive jump in AAMR at the onset of the pandemic can be attributed to the burden of the pandemic on CVD, sepsis, and limited healthcare system accessibility, which led to delays in diagnosis and treatment. Infection with SARS-CoV2 leads to an increase in myocardial injury, both in patients with and without pre-existing cardiovascular disease (15, 16). The SARS-CoV2 virus also increases proinflammatory cytokines and chemokines, unleashing a cytokine storm in the infected individual that can lead to multiple organ dysfunction (17, 18). Additionally, immunosuppression in the form of lymphopenia is implicated in severe COVID-19 and contributes to a dysregulated immune response and, ultimately, sepsis (19). Pathophysiology aside, the pandemic led to delays in outpatient healthcare access for patients with cardiovascular disease, increased rates of hospital avoidance, and fewer hospital admissions for those patients as resources were directed toward patients with acute infection (20–22).

With respect to gender, a similar pattern to overall mortality—a gentle decline in AAMR followed by a subsequent increase with the onset of the COVID-19 pandemic—is evident in both men and women during this period. Pandemic-era spikes are clearly present for both men and women, just as in the overall dataset. Male mortality remained consistently higher than female mortality throughout all the years encompassed in our analysis. Prior research indicates higher male sepsis-associated CVD mortality may be attributed to the higher risk of bloodstream infections in men, and to the higher rate of cardiovascular disease in men (23–25). In fact, it has been demonstrated that elevated mortality in men from bloodstream infections is at least partly mediated by cardiovascular risk factors (24).

We also identified racial disparities in AAMR, with non-Hispanic Black adults having the highest AAMR before and during the pandemic, as compared to NH White, Hispanic, and NH American Indian or Alaskan Native adults. In 1999, NH Black adults had an AAMR nearly twice as high as Hispanics and nearly triple that of White and American Indians or Alaskan Natives. In subsequent years, NH Black adults’ AAMR trended consistently downward until the beginning of the pandemic. These data trends align with those from prior studies of sepsis AAMR, which noted elevated mortality rates among Black adults but declining mortality in Black and Hispanic adults in the years before the COVID-19 pandemic (26). With the onset of the pandemic, AAMR in NH Black adults increased to a level such that the progress of decreasing AAMR over the previous 15 years was lost. The tangible impacts of the social determinants of health are evident in these disparities among Black adults. Higher incidence of comorbidities such as hypertension, diabetes, and asthma, and disparities in care access and outcomes, increase mortality related to CVD and sepsis in Black adults. Black adults were also found to be more likely to work high-risk frontline jobs during the pandemic that cannot be done remotely, and they are more likely to have limited access to healthcare and insurance (27).

The elderly population had a decreasing mortality trend compared to a stable trend for the middle-aged population before the COVID-19 pandemic. We found that the pandemic had a greater impact on the long-term trend for the elderly, with AAMR rising at double the rate of the middle-aged population during the pandemic years. Elderly adults have a greater susceptibility to infection by SARS-CoV2 due to their weaker immune defenses (28, 29). Elderly adults are also more likely to develop tissue damage from infection because of increased cytokine release due to COVID-19. When compared to middle-aged adults, elderly adults generally have more existing health conditions that increase the risk of CVD and sepsis-related mortality (29, 30).

Our investigation of state-level change in sepsis-associated CVD AAMR unearths the undeniable impact of the COVID-19 pandemic on mortality nationwide for patients with sepsis-associated cardiovascular disease. A moment's glance at the maps of AAMR from 1999 to 2019 and from 2019 to 2021 reveals the dramatic jump in mortality in all but three Northeastern states (Figures 6a,b). The near-even split from 1999 to 2019—with AAMR dropping in half of the states while rising in the other half—is disrupted during the three-year period encompassing the COVID-19 pandemic. Mapping and color-coding the AAMR data, as we have done, allows for further inspection of regional variation in AAMR change. Between 1999 and 2019, it appears that states with declining AAMRs are in the Northeast, Mid-Atlantic, and Southeast; all but five of these twenty-five states, as well as the District of Columbia (AAMR change of −84.61), are situated east of the Mississippi River. On this same map, the states with the most drastic sepsis-associated CVD AAMR increases are primarily clustered in the Midwest and Northwest. These geographic trends are upended in the map of AAMR change from 2019 to 2021, where all but three Northeastern states experienced a jump in AAMR. Seven of the ten states with the largest AAMR increases during this period—all but Wyoming (AAMR change of 39.99), California (27.42), and Delaware (24.57)—are located in the South Census Region (as classified by the CDC Wonder Multiple Cause of Death 1999–2020 dataset) (31). These findings align with prior research, which found that in the first full year of the pandemic, non-COVID-19 mortality in the Northeast increased less than would be expected given the concurrent COVID-19 mortality in those states (32). That same research, which also utilized the CDC WONDER database, found a greater-than-expected increase in non-COVID-19 mortality in the South census region.

Pandemic-era sepsis-associated CVD mortality varies clearly from state to state, likely due to varying levels of change in COVID-19 and non-COVID-19 mortality between states. Much media attention and scientific inquiry have been directed at state-level discrepancies in mortality during the pandemic. Research has linked statewide COVID-19 mortality rates with mean years of education, poverty rates, and healthcare access, factors which vary widely between US states (33). Links have also been drawn between COVID-19 mortality and levels of interpersonal trust, vaccination rates, vaccine allocation, and state-mandated lockdowns (33–36). Political partisanship (gauged by votes cast in the 2020 presidential election) and the urban-rural vaccination divide (although perhaps more applicable on a county level rather than a state level) are two additional factors which have been floated as underlying drivers of mortality discrepancies between states (33, 37, 38). Definitive conclusions will be challenging to draw. What appears certain is that the COVID-19 pandemic was accompanied by a substantial elevation in sepsis-associated CVD mortality in most states, even states that had seen declining mortality in the previous two decades.

This study provides valuable insight that can assist policy makers in improving healthcare interventions and shaping public health strategies. Identifying demographic disparities, such as the impact of race on sepsis-associated CVD mortality, highlights the importance of targeted interventions for vulnerable populations. Community health centers in underserved neighborhoods have been shown to increase access to healthcare and decrease poor heatlh outcomes (39, 40). By increasing funding for these centers, disadvantaged populations can have improved access to preventative healthcare, such as vaccinations, and more convienient access to treatment. Additionally, this disparities data can be used by policy makers to targeted regional improvements in healthcare equity. Expanding government insurance, such as Medicaid, as well as community-based efforts such as door-to-door vaccination education campaigns have the ability to increase coverage and health literacy across states with high mortality figures (33, 41). By integrating these findings into public health initiatives, policymakers can work to reduce health inequities, improve outcomes for patients with cardiovascular disease and sepsis, and build more resilient healthcare systems that are better equipped to manage future public health crises.

### Limitations

As our mortality data is based on information from death certificates, we are unable to rule out errors resulting from death certificate inaccuracies, which have been shown to impact national mortality databases (42). Interestingly, such inaccuracies may stem from pandemic-related changes in death reporting; a prior analysis utilizing the WONDER database suggested that the sepsis-related death toll in the first year of the pandemic may have been substantially underestimated (14). The nature of our study leaves us unable to determine the temporal association of septicemia and CVD—by counting records mentioning both causes of death, we are unable to know whether CVD resulted from preexisting septicemia or whether individuals with existing CVD developed septicemia. Though we note a marked elevation in mortality from 2020 to 2022, suggesting a pandemic-related cause, we cannot identify such a cause with certainty. Unfortunately, we were unable to include Asian or Pacific Islander adults in our analysis because of a sampling change in the CDC WONDER database in 2021. This change complicates temporal analysis of mortality in these individuals.

## Conclusion

Sepsis-associated CVD mortality has seen an increase across the US over the past decade, with the COVID-19 pandemic further exacerbating this trend. Significant disparities in trends have persisted throughout, with the elderly, men, NH Black adults, and the Southern region being disproportionately affected by the pandemic. This increase in mortality rates from sepsis and cardiovascular diseases and the worsening of disparities highlight the gaps in the healthcare system and the demographic and regional differences in healthcare accessibility during periods of strain. These findings also demonstrate the interconnected nature of chronic health conditions, infectious diseases, and the social determinants of health. To improve outcomes in all population groups, healthcare strategies must be developed that increase health equity throughout the patient population, especially in the context of public health crises like the COVID-19 pandemic.

## Data Availability

The original contributions presented in the study are included in the article/Supplementary Material, further inquiries can be directed to the corresponding author.
