# Supplementary material for: Trends in sepsis-associated cardiovascular disease mortality in the United States, 1999 to 2022
Source: Front Cardiovasc Med. 2024 Dec 9;11:1505905. doi: 10.3389/fcvm.2024.1505905 (PMC11663846; doi:10.3389/fcvm.2024.1505905)

Supplementary Table 1. Separate sepsis and cardiovascular disease related mortality in the United States.

Year	Sepsis only Deaths	Sepsis only AAMR	CVD only Deaths	CVD only AAMR
1999	129684	136.54	1358200	1432.56
2000	129246	134.27	1353258	1408.03
2001	131790	134.47	1344872	1375.33
2002	135038	135.57	1351995	1361.41
2003	137113	135.16	1342997	1328.35
2004	137868	133.69	1301587	1267.18
2005	144661	137.48	1316202	1254.43
2006	144551	134.57	1291793	1204.28
2007	144906	132.12	1282883	1170.17
2008	151593	135.28	1301295	1161.34
2009	149513	130.59	1275187	1113.94
2010	152395	130.89	1290537	1107.61
2011	157515	131.52	1307215	1089.61
2012	158059	128.75	1317406	1071.2
2013	166318	132.31	1350127	1071.97
2014	174686	135.59	1360607	1055.49
2015	187348	142.2	1411201	1070.16
2016	189663	140.88	1425324	1058.68
2017	195366	141.81	1473061	1069.77
2018	197530	140.21	1499022	1064.85
2019	192643	134.04	1516982	1056.86
2020	231703	158.29	1785073	1222.8
2021	247738	172.15	1812580	1283.05
2022	232597	155.64	1767275	1191.99
**Number of Joinpoints (years of Joinpoint)**		2 (2012, 2019)		2 (2011, 2018)
**APC-Segment 1 (95% CI)**		-0.27 (-4.25 to 4.45)		-2.46 (-4.20 to -1.94)
**APC-Segment 2 (95% CI)**		1.38 (-3.11 to 3.48)		-0.09 (-1.97 to 1.57)
**APC-Segment 3 (95% CI)**		4.85 (1.44 to 9.55)		4.36 (2.15 to 8.32)
**Average APC**		0.89 (0.46 to 1.28)		-0.58 (-0.87 to -0.34)

Supplementary Figure 1. Joinpoint models for separate sepsis and cardiovascular disease related mortality in the United States 1999 to 2022. *Indicates the APC is significantly different from 0.

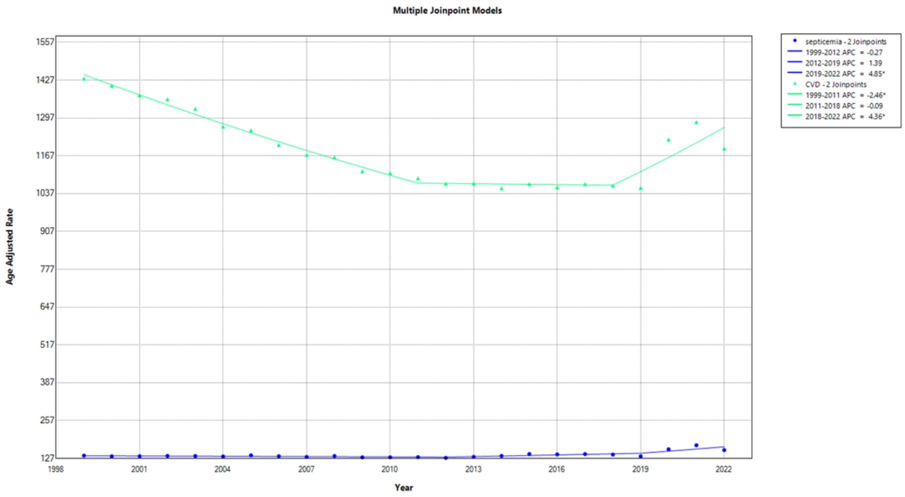

Supplementary Table 2. Sepsis-associated cardiovascular disease mortality rates stratified by gender in the United States.

	Age Adjusted Mortality Rate		
**Year**	**Overall**	**Female**	**Male**
1999	65.71	58.43	76.64
2000	64.64	57.42	75.23
2001	64.34	57.81	73.94
2002	64.32	57.39	74.57
2003	64.11	57.13	74.31
2004	62.79	55.92	72.86
2005	64.71	57.28	75.4
2006	62.55	55.43	72.73
2007	61.04	53.6	71.7
2008	61.48	54.51	71.41
2009	58.73	51.77	68.56
2010	58.42	51.28	68.41
2011	58.66	51.76	68.19
2012	57.33	50	67.38
2013	58.79	51.49	68.72
2014	59.79	52.31	69.87
2015	63.1	54.71	74.44
2016	63.18	54.43	74.78
2017	63.92	54.94	76
2018	63.86	54.5	76.19
2019	61.4	52.1	73.65
2020	73.07	60.81	88.82
2021	80.56	67.47	97.1
2022	74.33	62.87	89.59
**Number of Joinpoints (years of Joinpoint)**	1 (2013)	2 (2012, 2019)	1 (2013)
**APC-Segment 1 (95% CI)**	-1.02 (-2.13 to -0.33)	-1.11 (-3.57 to 0.14)	-0.96 (-2.05 to -0.22)
**APC-Segment 2 (95% CI)**	3.14 (2.01 to 5.40)	1.25 (-1.93 to 2.88)	3.56 (2.45 to 5.75)
**APC-Segment 3 (95% CI)**		6.13 (2.59 to 10.82)	
**Average APC**	0.59 (0.23 to 0.97)	0.52 (0.17 to 0.81)	0.79 (0.42 to 1.18)

Supplementary Figure 2. Joinpoint models for sepsis-associated cardiovascular disease mortality rates stratified by gender in the United States from 1999 to 2022. *Indicates the APC is significantly different from 0.

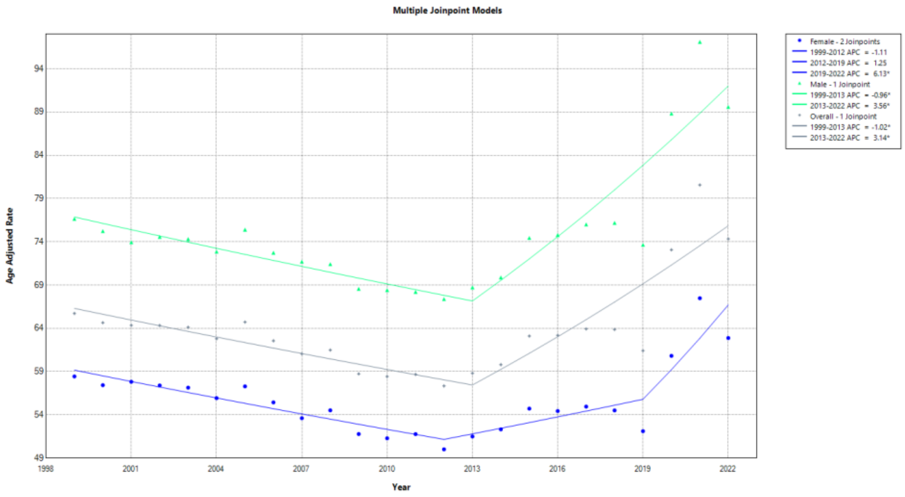

Supplementary Table 3. Sepsis-associated cardiovascular disease mortality rates stratified by race in the United States.

	**Age Adjusted Mortality Rate**			
**Year**	**NH White**	**NH Black**	**NH American Indian or Alaskan Native**	**Hispanic**
1999	57.62	139.54	68.45	77.72
2000	56.83	136.64	66.47	74.04
2001	56.4	134.98	55.98	78.17
2002	56.53	132.73	72.72	76.39
2003	56.77	130.59	70.87	73.9
2004	55.64	127.02	60.55	73.4
2005	57.31	129.54	62.47	77.86
2006	55.73	121.22	62.6	73.51
2007	54.37	118.94	69.71	70.35
2008	55.42	113.82	70.57	69.06
2009	52.95	107.74	77.79	64.75
2010	52.89	103.04	70.82	66.58
2011	53.61	99.99	73.76	65.46
2012	52.5	95.83	72.69	63.3
2013	53.76	98.2	72.77	64.87
2014	55.17	96.22	77.48	65.29
2015	58.74	99.5	79.87	66.24
2016	58.99	97.91	82.21	65.96
2017	60.02	96.38	84.9	66.47
2018	60.06	97.6	77.95	64.72
2019	58.31	91.91	74.45	60.41
2020	66.2	117.57	92.28	85.29
2021	75.06	123.02	100.06	88.25
2022	70.19	115.89	95.56	75
**Number of Joinpoints (years of Joinpoint)**	2 (2012, 2019)	1 (2017)	1 (2019)	1 (2017)
**APC-Segment 1 (95% CI)**	-0.62 (-2.67 to 0.79)	-2.39 (-3.00 to -1.88)	1.35 (-4.46 to 18.90)	-1.11 (2.47 to -0.29)
**APC-Segment 2 (95% CI)**	2.17 (-1.85 to 3.40)	6.09 (3.21 to 11.50)	7.15 (1.18 to 14.72)	5.96 (2.30 to 14.95)

**APC-Segment 3 (95% CI)**	6.22 (3.09 to 10.54)			
**Average APC**	1.10 (0.75 to 1.38)	-0.60 (-1.03 to -0.20)	2.09 (1.15 to 3.31)	0.39 (-0.27 to 1.05)

Supplementary Figure 3. Joinpoint models for sepsis-associated cardiovascular disease mortality rates stratified by race in the United States from 1999 to 2022. *Indicates the APC is significantly different from 0.

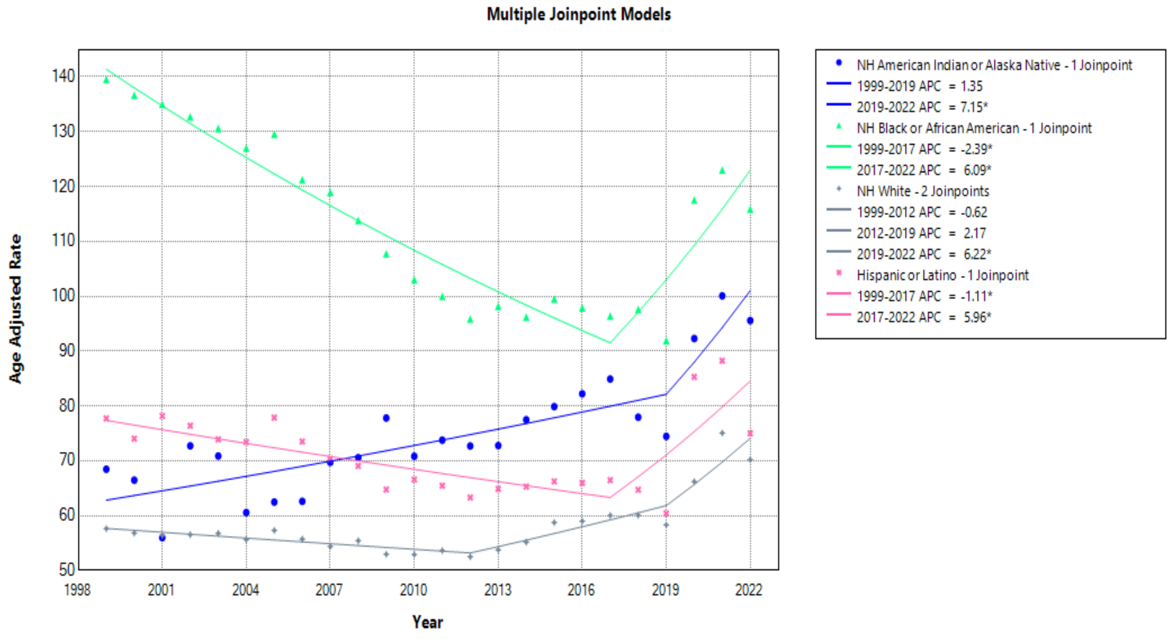

Supplementary Table 4. Sepsis-associated cardiovascular disease mortality rates stratified by age-group in the United States.

	Age Adjusted Mortality Rate	
**Year**	**Middle Age (45-64y)**	**Elderly (65y+)**
1999	15.62	153.73
2000	15.66	150.7
2001	15.	149.47
2002	15.48	150.13
2003	15.7	149.17
2004	15.43	146.03
2005	15.68	150.85
2006	15.63	145.02
2007	15.13	141.72
2008	15.43	142.4
2009	15.09	135.41
2010	14.81	135.04
2011	15.27	134.9
2012	15.07	131.59
2013	16.01	133.96
2014	16.84	135.25
2015	17.5	143.22
2016	17.93	142.7
2017	17.89	144.8
2018	18.57	143.44
2019	18.19	137.33
2020	23.98	159.35
2021	27.6	173.63
2022	24.03	162.73
**Number of Joinpoints (years of Joinpoint)**	2 (2011, 2018)	2 (2012, 2019)
**APC-Segment 1 (95% CI)**	0.39 (-6.31 to -6.42)	-1.13 (-3.60 to 0.97)
**APC-Segment 2 (95% CI)**	3.13 (-3.14 to 6.69)	1.34 (-2.52 to 2.72)
**APC-Segment 3 (95% CI)**	9.46 (4.91 to 17.46)	5.37 (2.21 to 9.56)
**Average APC**	2.33 (1.67 to 2.93)	0.45 (0.11 to 0.72)

Supplementary Figure 4. Joinpoint models for sepsis-associated cardiovascular disease mortality rates stratified by age-group in the United States from 1999 to 2022. *Indicates the APC is significantly different from 0.

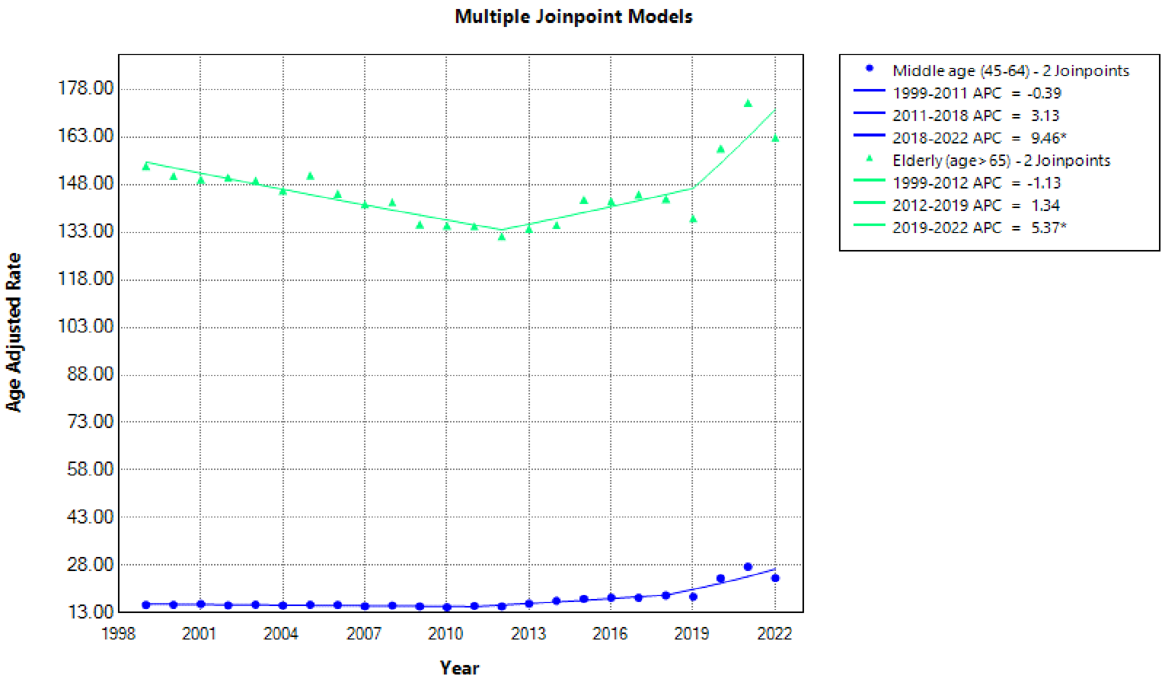

Supplementary Table 5. Sepsis-associated cardiovascular disease mortality rates stratified by geographic region in the United States.

	Age Adjusted Mortality Rate			
**Year**	**Northeast**	**Midwest**	**South**	**West**
1999	79.44	53.7	70.61	56.94
2000	79.51	52.09	69.69	54.84
2001	78.06	52.16	68.69	56.75
2002	77.23	51.8	68.97	57.28
2003	75.12	51.18	69.41	58.4
2004	75.35	50.53	65.99	58.37
2005	77.34	52.52	68.98	58.31
2006	74.76	49.81	65.62	59.21
2007	72.17	49.13	64.3	57.66
2008	70.4	50.52	64.78	59.07
2009	64.34	48.51	62.93	57.02
2010	65.52	47.19	61.49	58.22
2011	65.48	48.31	60.97	59.08
2012	63.19	46.98	60.14	57.75
2013	64.94	47.48	61.65	59.81
2014	66.33	48.51	63.39	58.9
2015	67.54	53.02	66.78	62.79
2016	65.56	51.85	67.26	65.18
2017	63.06	53.32	69.13	66.16
2018	61.72	53.56	70.67	63.83
2019	59.02	53.62	66.83	61.44
2020	68.87	63.84	80.12	73.09
2021	69.67	67.15	91.91	82.86
2022	68.91	62.34	82.18	76.67
**Number of Joinpoints (years of Joinpoint)**	1 (2018)	1 (2013)	1 (2013)	1 (2019)
**APC-Segment 1 (95% CI)**	-1.39 (-1.81 to -1.09)	-0.90 (-1.56 to -0.37)	-1.22 (-2.37 to -0.43)	0.78 (0.29 to 1.16)
**APC-Segment 2 (95% CI)**	3.54 (0.81 to 9.13)	3.63 (2.75 to 4.93)	3.98 (2.77 to 6.15)	8.60 (4.06 to 15.20)
**Average APC**	-0.55 (-0.89 to -0.27)	0.85 (0.58 to 1.13)	0.78 (0.39 to 1.20)	1.76 (1.35 to 2.13)

Supplementary Figure 5. Joinpoint models for sepsis-associated cardiovascular disease mortality rates stratified by geographic region in the United States from 1999 to 2022. *Indicates the APC is significantly different from 0.

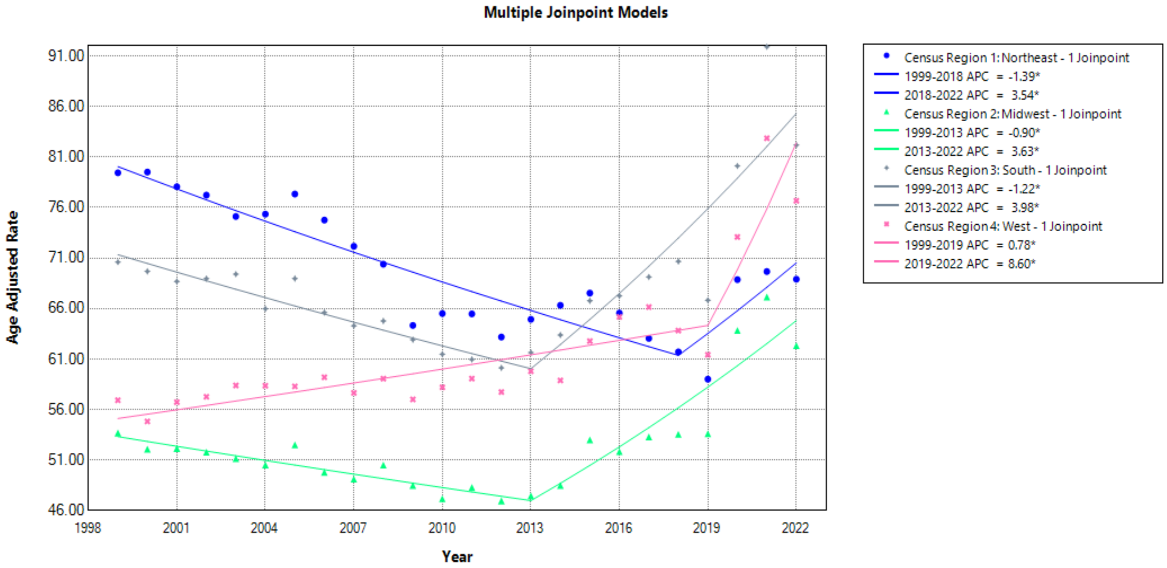

Supplementary Table 6. Sepsis-associated cardiovascular disease mortality rates stratified by state in the United States.

	**Age Adjusted Mortality Rate**				
**State**	**1999**	**2019**	**2021**	**Change in AAMR per 100,00 from 1999 to 2019**	**Change in AAMR per 100,00 from 2019 to 2021**
Alabama	72.55	70.63	104.5	-1.92	33.87
Alaska	46.9	49.41	52.4	2.51	2.99
Arizona	34.75	29.71	45.6	-5.04	15.89
Arkansas	67.47	85.79	106	18.32	20.21
California	74.29	76.08	103.5	1.79	27.42
Colorado	34.03	48.86	70.3	14.83	21.44
Connecticut	70.57	64.56	57.7	-6.01	-6.86
Delaware	73.39	37.43	62	-35.96	24.57
District of Columbia	177.09	92.48	108.6	-84.61	16.12
Florida	51	51.35	74.8	0.35	23.45
Georgia	81.52	68.45	88.6	-13.07	20.15
Hawaii	66.29	35.07	40.3	-31.22	5.23
Idaho	30.28	45.97	58.5	15.69	12.53
Illinois	62.79	50.6	57.2	-12.19	6.6
Indiana	55.48	70.22	84.1	14.74	13.88
Iowa	37.28	53.42	60.9	16.14	7.48
Kansas	42.42	53.29	73.1	10.87	19.81
Kentucky	68.07	99.48	132.4	31.41	32.92
Louisiana	79.03	62.73	89.6	-16.3	26.87
Maine	44.11	19.48	15.9	-24.63	-3.58
Maryland	104.86	63.49	86.8	-41.37	23.31
Massachusetts	61.73	58.6	65.4	-3.13	6.8
Michigan	63.18	51.83	67.5	-11.35	15.67
Minnesota	23.71	48.23	63.2	24.52	14.97
Mississippi	81.43	94.46	139.7	13.03	45.24
Missouri	51.35	45.57	61.2	-5.78	15.63
Montana	26.13	48.65	71.5	22.52	22.85
Nebraska	35.3	60.01	78.2	24.71	18.19
Nevada	87.35	82.43	106.2	-4.92	23.77
New Hampshire	43.35	44.08	46.5	0.73	2.42
New Jersey	100.79	75.29	90.4	-25.5	15.11
New Mexico	36.77	47.74	63.9	10.97	16.16

New York	93.97	60.24	70.6	-33.73	10.36
North Carolina	72.42	54.51	72.4	-17.91	17.89
North Dakota	26.9	53.56	69.5	26.66	15.94
Ohio	73.49	56.24	73.8	-17.25	17.56
Oklahoma	59.64	117.2	154.6	57.56	37.4
Oregon	27.33	42.03	51.5	14.7	9.47
Pennsylvania	67.08	52.49	71.2	-14.59	18.71
Rhode Island	67.04	60.88	55.4	-6.16	-5.48
South Carolina	80.96	76.62	99.6	-4.34	22.98
South Dakota	30.66	71.51	81	40.85	9.49
Tennessee	74.45	67.72	98.3	-6.73	30.58
Texas	74.85	75.11	102.7	0.26	27.59
Utah	36.46	45.76	55.3	9.3	9.54
Vermont	50.29	44.04	49.1	-6.25	5.06
Virginia	63.5	43.05	59.6	-20.45	16.55
Washington	34.32	58	73.7	23.68	15.7
West Virginia	82.85	90.91	113.5	8.06	22.59
Wisconsin	33.73	48.63	60.1	14.9	11.47
Wyoming	39.5	35.41	75.4	-4.09	39.99

Supplementary Figure 6a. Trends in sepsis-associated cardiovascular disease mortality, stratified by state, in the United States between 1999 and 2019.

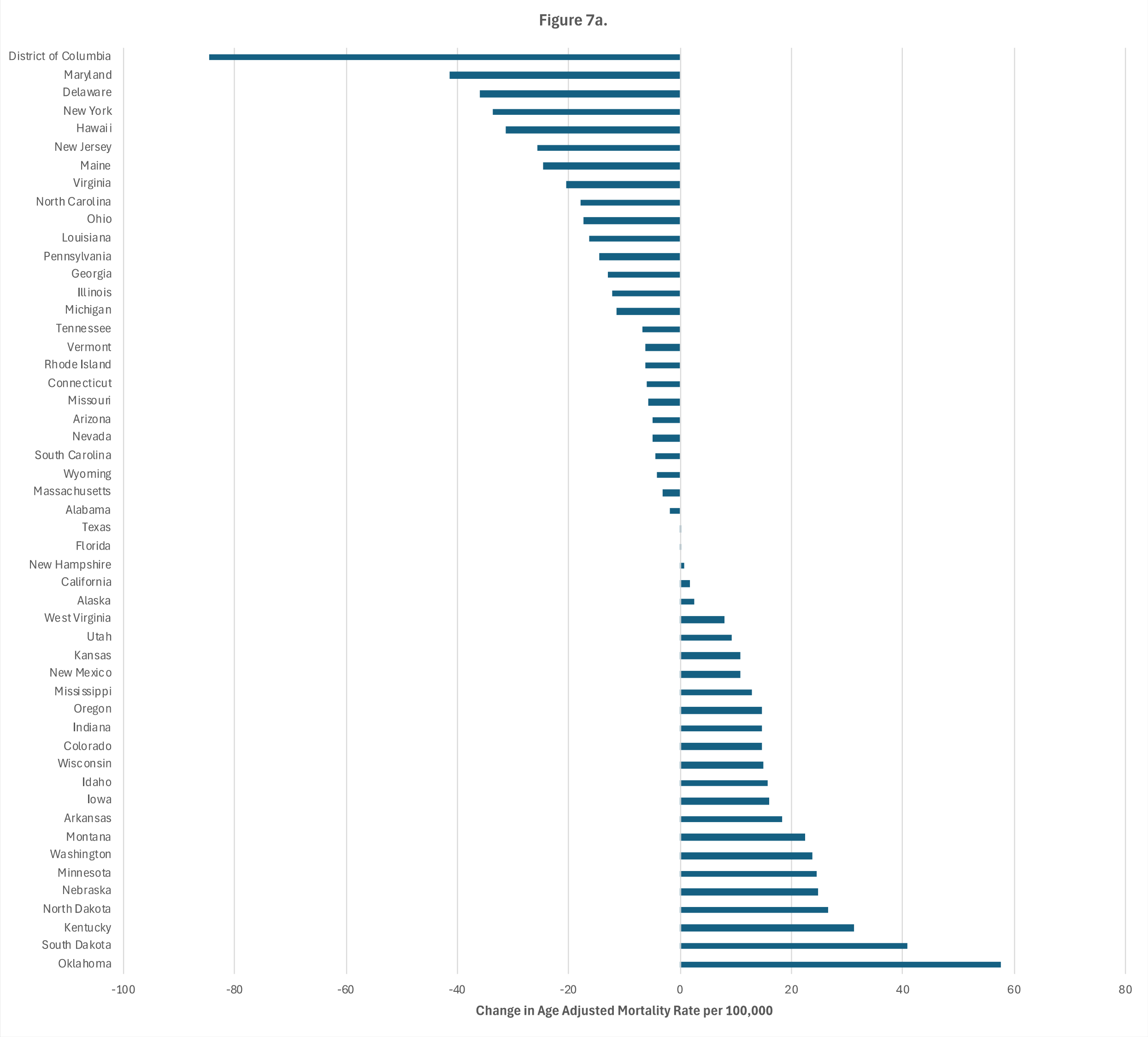

Supplementary Figure 6b. Trends in sepsis-associated cardiovascular disease mortality, stratified by state, in the United States between 2019 and 2021.

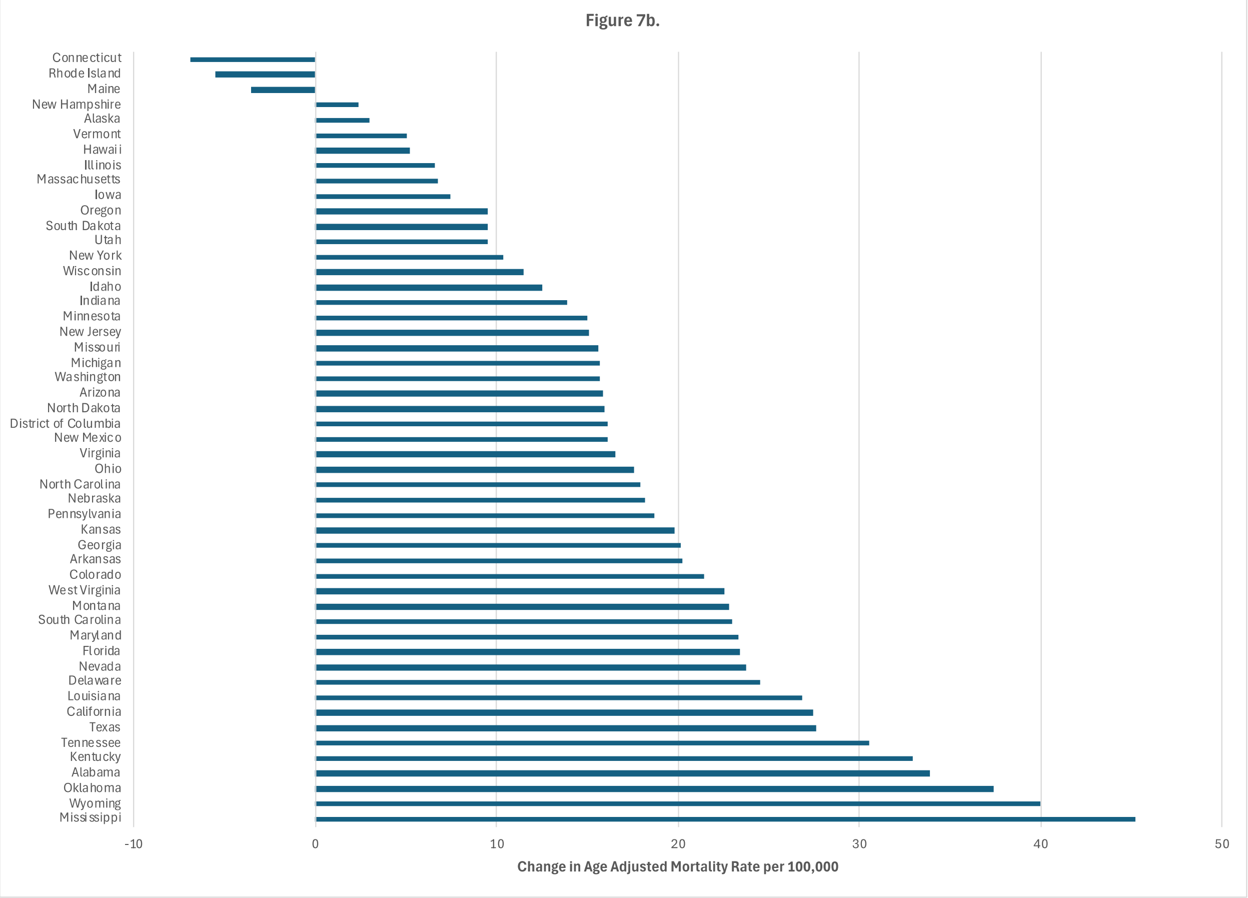

Supplement: Supplementary file 1 [file Datasheet1.pdf]
